# Evaluation of the Photoplethysmogram-Based Deep Learning Model for Continuous Respiratory Rate Estimation in Surgical Intensive Care Unit

**DOI:** 10.3390/bioengineering10101222

**Published:** 2023-10-19

**Authors:** Chi Shin Hwang, Yong Hwan Kim, Jung Kyun Hyun, Joon Hwang Kim, Seo Rak Lee, Choong Min Kim, Jung Woo Nam, Eun Young Kim

**Affiliations:** 1 Spass Inc., 905Ho, RnD Tower, 396, Worldcup Buk-ro, Mapo-gu, Seoul 03925, Republic of Korea; noritheyellow@gmail.com (C.S.H.); jw.nam@spass.ai (J.W.N.); 2 Division of Trauma and Surgical Critical Care, Department of Surgery, Seoul St. Mary’s Hospital, College of Medicine, The Catholic University of Korea, Banpo-daero 222, Seocho-gu, Seoul 06591, Republic of Korea

**Keywords:** photoplethysmogram, respiratory rate, intensive care unit, surgery, signal, prediction, deep learning, convolutional neural network, residual neural network

## Abstract

The respiratory rate (RR) is a significant indicator to evaluate a patient’s prognosis and status; however, it requires specific instrumentation or estimates from other monitored signals. A photoplethysmogram (PPG) is extensively used in clinical environments as well as in intensive care units (ICUs) to primarily monitor peripheral circulation while capturing indirect information about intrathoracic pressure changes. This study aims to apply and evaluate several deep learning models using a PPG for the continuous and accurate estimation of the RRs of patients. The dataset was collected twice for 2 min each in 100 patients aged 18 years and older from the surgical intensive care unit of a tertiary referral hospital. The BIDMC and CapnoBase public datasets were also analyzed. The collected dataset was preprocessed and split according to the 5-fold cross-validation. We used seven deep learning models, including our own Dilated Residual Neural Network, to check how accurately the RR estimates match the ground truth using the mean absolute error (MAE). As a result, when validated using the collected dataset, our model showed the best results with a 1.2628 ± 0.2697 MAE on BIDMC and RespNet and with a 3.1268 ± 0.6363 MAE on our dataset, respectively. In conclusion, RR estimation using PPG-derived models is still challenging and has many limitations. However, if there is an equal amount of data from various breathing groups to train, we expect that various models, including our Dilated ResNet model, which showed good results, can achieve better results than the current ones.

## 1. Introduction

The respiratory rate (RR) can be useful for critically ill patients as a significant indicator to evaluate a patient’s prognosis and status, as it not only represents a clinical status change in a patient’s respiratory system function but also participates in the respiratory compensation mechanism for tissue hypoperfusion and systemic circulation dysfunction such as shock [1,2]. The importance of the RR can also be seen in the various scoring systems such as the Acute Physiology And Chronic Health Evaluation (APACHE) [3], which assesses the prognosis of a patient’s disease in the intensive care unit (ICU); the Sequential Organ Failure Assessment (SOFA, qSOFA) [4], which conventionally assesses the level of organ failure and infection; and the Modified Early Warning Score (MEWS) [5], which is used for the continuous surveillance of the patient’s condition. However, the general RR measuring method that is currently conducted in clinical practice is a manual counting method measured by nursing staff, which does not fit for continuous all-time surveillance [6]. Although various RR estimation methods using physiological signals (etCO2, ECG, Impedance Pneumography signal, and Oral–Nasal Pressure) have been developed to compensate for this shortcoming [7,8,9], a requirement of additional equipment such as an impedance meter, ECG leads, the restriction of movement due to such equipment, and the difficulty of always-on measurement come as its limitations. Eventually, this limits the applicability of these methods as standard methods for continuous real-time RR surveillance in critically ill patients.

A photoplethysmogram (PPG) is a physiological signal that implies the information of blood volume changes in the microvascular bed of tissue detected using the pulse oximeter. It is normally acquired from a subject’s peripheral site such as a fingertip, with a light-emitting diode that illuminates a red or a near-infrared wavelength and detects its reflections through the photodetector. At this point, the most affecting factor of the signal is the blood volume, especially in the arteries, as the blood vessel constantly changes in response to cardiac contraction, breathing, and the autonomic nervous system [10,11]. Therefore, the morphological characteristics and time-dependent attributes of the PPG signal contain various and significant clinical information that indirectly indicates a subject’s heart rate, blood pressure, oxygen saturation, and respiratory rate [11,12].

Deep learning (DL), an Artificial Intelligence algorithm that is widely utilized in image detection, time forecasting, natural language processing, etc., according to the architectural design, can be trained to extract various features effectively from the physiological signal (e.g., PPG) using a feature extractor [13] and to estimate another signal (e.g., RR). Herein, we collected real-time PPG and RR data from patients admitted to the surgical intensive care unit (SICU) of a tertiary referral hospital in Korea, and they were used in several previously studied DL models for RR estimation. Moreover, we implemented a novel DL model and compared its performance with others to verify the significance of a PPG-derived DL model’s RR estimation.

## 2. Materials and Methods

### 2.1. PPG Measurement and Dataset Collection

Our dataset in the current study consisted of 100 patients aged 18 years and older. Experiments were conducted in the SICU of our institution, which has been operated as a closed ICU system by two critical care specialists who take care of all patients admitted to the SICU. The diagnosis of a patient was made through a consultation with the specialists, and the decision was made by considering the patient’s blood test, physical examination, imaging test, and history. The dataset contained demographic information, PPG signals, and exhalation timestamps for each de-identified subject. Two-minute PPGs were collected for each subject twice, sampled at 125 Hz. A unique ID was generated for each collected two-minute PPG. This ID served as an identifier for individual data and as a de-identifier for the subject at the same time. Table 1 shows the participants’ characteristics and the details extracted from our dataset.

**Figure 1:**
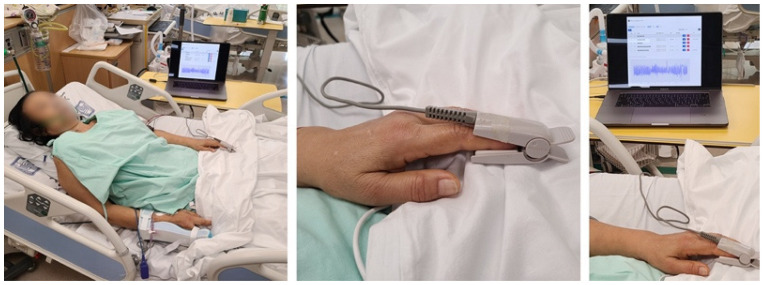
Picture of PPG collecting process.

The PPG signal collector is a respiratory counter web application, and its signal collecting process is represented in Figure 1. A router was linked with a laptop to connect the network between the browser and the patient monitor. In this study, for our research, data were collected from patients through a pulse oximeter connected to the patient’s monitor. All patient measurements were taken while they were lying in the supine position. The subject’s demographic information must be entered into the PPG signal collector to acquire the data. When the collection started, the PPG signal was continuously recorded during the time limit, and the RR was recorded manually by medical staff pressing the spacebar at the exhalation time. To ensure the collection of 2 min of data, the measurement time was set to 2 min and 10 s. When the time ended, the data were recorded and stored in the database. Medical staff supported our data collection using the PPG signal collector. 

The details of the collection procedure are as follows:

1. Move into the local monitoring page through the browser installed on the laptop.
2. Set up the measuring time.
3. Register the subject’s demographic information including gender, age, and diagnosis to start recording.
4. When a window pops up, click the start button to record the PPG.
5. Press the spacebar when the subject exhales.
6. When the time is up, the window will close, and the data will be stored automatically.

**Figure 2:**
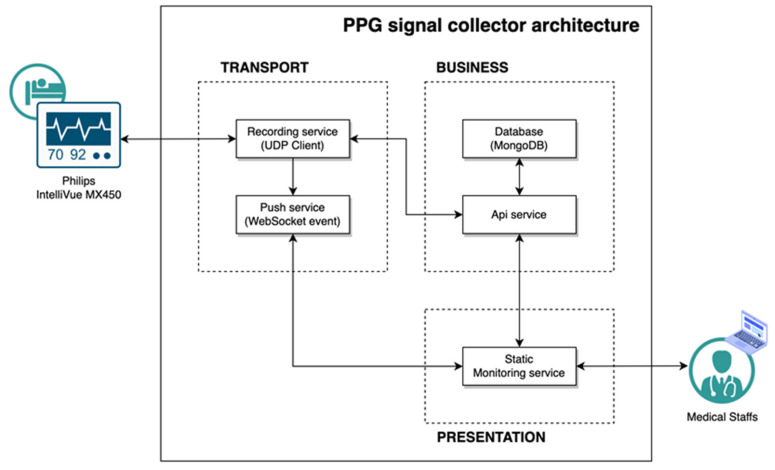
Signal collector architecture.

The PPG signal collector receives medical data from the Philips IntelliVue MX450, referring to the interlocking specification provided by Philips [14]. We used a MacOS Big Sur v11.4 laptop with a 1.3 GHz dual-core Intel Core 7 processor CPU and 8 GB 1600 MHz DDR3 memory for our research and used Safari v14.11 for the browser. The application was developed in NestJS v8.0.0 and Vue v3.2.31 based on Javascript 2017 (ES8), and the database was configured with MongoDB v5.0.9. The overall structure of the PPG signal collector is shown in Figure 2. All research protocols followed were in accordance with the ethical standards of the responsible committee on human experimentation and with the Helsinki Declaration of 1975, as revised in 2000. All data used in this study were anonymized, deidentified, and aggregated before analysis. Informed consent was obtained from all participants or caregivers. This study was approved by the Institutional Review Board of the Ethics Committee of Seoul St. Mary’s Hospital (IRB No. KC21ONSI0839).

### 2.2. Additional Dataset

In addition to the above datasets, we used additional datasets for the training and validation of DL models. The datasets are described below.

#### 2.2.1. BIDMC

BIDMC [15,16] is 53 samples of 8 min duration signals collected from ICU of Beth Israel Deaconess Medical Center. Two annotators labeled the RR using each subject’s impedance respiratory signal. However, we excluded samples with IDs 13, 15, and 19, which had missing values for RR, so a total of 50 samples were used in the study. The dataset consists of impedance respiratory, electrocardiogram, and PPG sampled in 125 Hz. It also includes demographics and 1 Hz signals such as heart rate, respiratory rate, O_2_ saturation, and pulse. This dataset provides various formats, including csv, for countless research and benchmark tests, and it is a public dataset. In this study, we used the PPG signal and RR provided by the dataset to validate the model.

#### 2.2.2. CapnoBase

The IEEE TBME Respiratory Rate Benchmark dataset [17] is a dataset designed for developing and testing RR estimation. The dataset contains 8 min electrocardiogram, capnography, and PPG signals acquired from 42 patients during elective surgery and routine anesthesia. Labels from an annotator are available for peaks from PPG and breaths from CO_2_. The data were obtained from 29 children and 13 adults. The dataset was used for validation purposes, and only 13 adult patients were used in this study, given that the study was conducted on adult patients.

### 2.3. Preprocessing

When measuring the PPG signal using a pulse oximeter, various low- and high-frequency noises that become biases in model training are generated because of motion artifacts, probe–tissue interface disturbance, powerline interference due to the instrumentation amplifiers, and changes in physiological parameters such as cardiac impulse [11]. Therefore, removing such noises lurking in the signal using signal filtering is a mandatory process, and it can improve the estimation accuracy of DL models effectively. We filtered the signal using a Hamming window and applied Finite Impulse Response (FIR) band-pass filter (BPF) with a cut-off frequency ranging from 0.1 to 0.4 Hz [18,19,20,21]. This is a frequency band that corresponds to respiration, and several studies have suggested an arbitrary cut-off between 0.05 and 0.4 Hz for estimating the RR [16,20,22,23]. Then, the filtered signal, in turn, was sliced into data samples of constant intervals for a fitted size to train the DL model. An interval of 60 s with 1 s shifting was set to slice the signal, as the RR, which we were trying to estimate, is conventionally counted per minute. Through the process above, we generated 6508, 21,050, and 5473 samples of 1 min PPG and RR values (brpm) from our self-collected dataset, BIDMC, and CapnoBase, respectively. Additionally, to enhance the efficiency of model calculation, a signal of 125 Hz was resampled into 30 Hz using linear interpolation [24,25]. The described preprocessing method applies to all of the datasets introduced above.

### 2.4. Deep Learning Models

For the next step, our patient’s preprocessed dataset was exploited to train the DL models, which we introduce below: Residual Neural Network, U-Net, Long Short-Term Memory, and Dilated Residual Neural Network. Each model has been utilized in many studies, using PPG to estimate the RR or its reference signal [25,26,27]. Except for the Dilated Residual Neural Network we are proposing, the remaining six models were implemented directly by referring to the architecture and parameters of each of the introduced papers, and the related code can be found at the following link: https://github.com/Noritheyellow/Project-RRpo-2ndStudy (accessed on 12 October 2023.).

#### 2.4.1. Residual Neural Network (ResNet)

ResNet, as a DL model introduced to overcome the vanishing gradient problem that occurs as the layers of backpropagation-based models become substantially deeper, takes the idea of summing the input and output values of the layer [28]. This is because the gradient, which has an important effect on the learning of weights in error propagation-based models, converges to zero as the layer becomes deeper, resulting in sluggish learning. To overcome this problem, ResNet simply sums the input and output values of an arbitrary layer. With ResNet, you can prevent learning by gradually losing the original shape and features of the PPGs due to the feature extraction process of data using PPGs and the pooling process that reduces the size of the data. As in the case of Bian [25], we implemented a ResNet block that reduces the size of the PPG using a convolutional layer and extracts features by accumulating them, and we implemented the model by applying it five times and used it to predict the RR value. CapnoBase, BIDMC, and synthetic datasets were used as the datasets, and the MAE was about 3.8 ± 0.5 when only real-world data were trained, and the result was about 2.5 ± 0.6 when synthetic data were mixed. In this paper, we implemented the model according to its description, and used a CapnoBase, BIDMC, and tertiary referral hospital data with it.

#### 2.4.2. U-Net

U-Net is a DL model designed to solve the pixel-wise labeling of images in biomedical image segmentation [29]. To prevent the loss of characteristics in the whole image context, the model extracts the features from each downsampling step and concatenates them to other extracted features, i.e., upsampled features. U-Net performs the above process through a set of layers called the contracting path. At this time, the features of each stage extracted from the contracting path are concatenated with the feature map of the symmetrical expanding path. The features are then upsampled using convolution to convert them to a higher resolution and then propagated to the next layer so that they can be combined with the features of the contracting path. The architecture of this overall model is U-shaped, hence the name.

Ravinchandran [26] proposed a model called RespNet, which is based on U-Net and uses a Dilated Residual Inception Block internally. The model trains the PPG to predict RR reference signals such as Capnometry, Impedance Pneumography, and the Oral–Nasal Pressure signal of a subject and uses Capnobase and Vortal datasets for this purpose. The prediction performance of the model is 0.262, and there is a 0.145 MSE for each dataset, as shown in the paper, but the cost burden and difficulty in securing patients may be a limitation because respiratory signals rather than RRs must be collected as a reference. Note that it is the RR value, and not the RR reference signal, that we wanted to estimate in this study, so we used a dense layer to change its output for that purpose.

#### 2.4.3. Long Short-Term Memory (LSTM)

LSTM, as a DL model designed to address the problem of forgetting the extracted features from the sequential data, i.e., the vanishing gradient problem that traditional recurrent neural network (RNN) models challenge, involves a computational process that selectively keeps only the necessary features over time [30]. Because of these properties, LSTMs are often used as linguistic models, but they have also shown significant results for one-dimensional data such as signals. Since such a feature of LSTM is useful for sequential data estimation, various models were implemented upon it.

Convolutional-LSTM [31] combines an LSTM that extracts temporal features from the incoming sequence data with a convolutional layer that extracts spatial features to provide a more holistic view of the data. Bidirectional LSTM [32] introduces an additional LSTM that trains in the backward direction and uses sequence data is the input, as opposed to the traditional LSTM that trains only in the forward direction. This approach is expected to yield better results because it obtains information from both the forward and backward flows of time and uses it for prediction. Attention [33], which mimics the cognitive action of attending to specific features, has been shown to significantly improve the prediction performance of sequential data, such as linguistic sentences, and is often used for signals that are also sequential data. Models that use attention mechanisms identify their own contextual relationships and utilize them for prediction. In addition to the most basic dot product attention, Bahdanau attention, entangled attention, and quantum attention [34,35,36] are used to measure the similarity between these data.

Kumar [27] tried to predict the RR by training vital signs from Capnobase, BIDMC, and sEMG datasets with various LSTM models such as LSTM, bidirectional LSTM (Bi-LSTM), Attention-based Bi-LSTM, CNN-LSTM, etc. However, the paper had limitations in terms of practicality because it used signals such as ECG and sEMG to predict the RR instead of using PPG alone. In this study, we implemented all models from the bottom and set parameters according to the paper described. The units of the models, specifically Vanilla LSTM, CNN LSTM, Bi-LSTM, and Attention-based Bi-LSTM, were modified into 256, 256, 128, and 64.

#### 2.4.4. Dilated Residual Neural Network (Our Proposed Model)

In this study, we did not only estimate the RR using the PPG-derived DL models that we introduced above, but we also used the model that we implemented, a Dilated Residual Neural Network (Dilated ResNet). The implemented model was layered using a dilated convolutional layer [37,38] additionally, and the inputs and outputs of each layer group were summed to overcome the vanishing gradient problem. First, the input PPG signal was passed through each of the 3 horizontally organized dilated convolutional layers. This allowed the model to characterize the signal’s relationships between data that are not only adjacent but also temporally spaced apart, facilitating the capture of the morphological features of the overall PPG. In addition, we tried to extract more characteristic information from the existing signal by adding another vertically identical layer. At this point, all features extracted from the layer group and feature-wise average values of input data were summed and used as the output. This process works inside a single unit block, the RespBlock, where the feature extraction of the RespBlock is followed by downsampling on its result. This process of the DL model that we considered compresses the existing extracted features densely and reduces the required computational resources for learning, so it continuously repeats the feature extraction and compression. Our study processed feature extraction and compression three times for each sample and doubled the output feature size for each iteration. Then, we applied the average pooling layer and fully connected layer on the output to estimate the final RR value.

**Figure 3:**
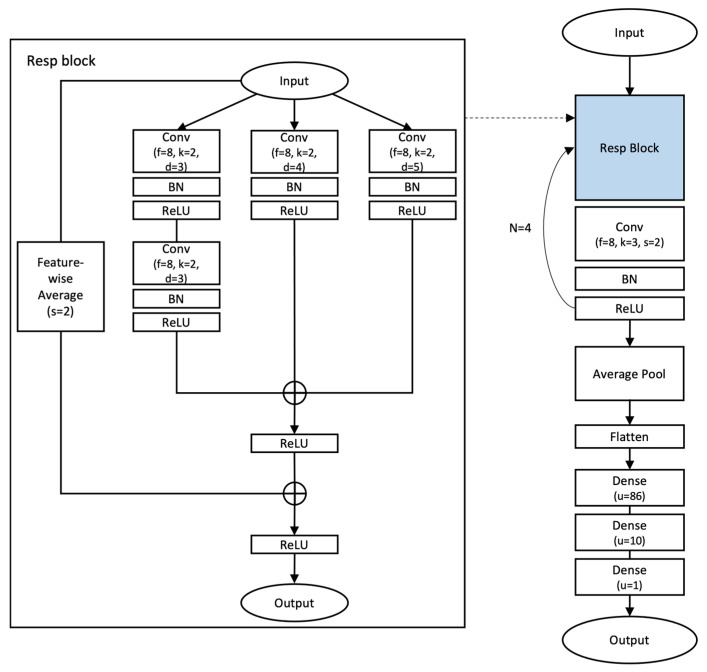
Dilated ResNet architecture.

For the proposed DL model to show good results, it is important to find the optimal hyperparameters. In the Dilated ResNet model, the hyperparameters are the number of RespBlocks ($N_{blk}$), kernel size of the convolutional layer inside the RespBlock ($kernel_{blk}$), dilation rate of the convolutional layer inside the RespBlock ($D_{blk}$), kernel_size of 1D convolutional layer for downsample ($kernel_{dwn}$), number of filters multiplied by the power of two ($filters_{c}$), stride size of average pooling ($S_{c}$), and units of first dense layer ($n_{den}$), and we tried to estimate the RR accurately by adjusting these parameters. To select the optimal hyperparameters, we used the Bayesian optimization algorithm [39], which has been used in studies of various models. Table 2 summarizes each of these hyperparameters and their experimental values, and the final selected parameters and model structure can be seen in Figure 3.

### 2.5. Experiments

In this study, we trained and validated the models using only our datasets to understand the performance of each model introduced above when trained on datasets collected from real-world clinics. To evaluate the generalization performance of the trained models, we extracted some unseen data from the self-collected datasets and added the BIDMC and CapnoBase datasets to form a test dataset. Additionally, BIDMC was utilized as a training and validation dataset to compare the RR estimation performance of various models, including the model proposed in this study. All trained models were evaluated using the authors’ dataset, CapnoBase, and BIDMC pre-segmented test datasets. Lastly, since the purpose of this study was to estimate the RRs of critically ill patients, we further tested and evaluated the robustness of the model to motion artifacts. Therefore, we extracted signals from random patients from the BIDMC test dataset and added artificial baseline wanderings created by varying the amplitude and frequency to those signals to produce signals with signal-to-noise ratios (SNRs) of 20 db, 15 db, and 10 db, respectively. These signals were intended to reflect motion artifacts, such as high-intensity accelerations, which affect PPG signals [40].

#### 2.5.1. Training and Validation method

Two datasets were used to train and validate the model: the authors’ own dataset and BIDMC. In order to perform independent training and validation of the model, we tried to completely distinguish the training and validation datasets according to the patient ID, so we performed randomly shuffled 5-fold cross-validation based on patient ID. All PPG signals and ground truth RR values were configured with a batch size of 256. The starting learning rate was set to 0.001 and optimized using Adam optimization [41]. Training was performed over 1000 epochs. In addition, if the loss value for the validation dataset formed a plateau with no change for a certain number of epochs when the model was learning, the learning rate was increased by a factor of 0.1 for more precise learning, and an early stopping technique was applied at the learning stage to prevent overfitting. All the models introduced here were implemented with Tensorflow version 2.0.

#### 2.5.2. Evaluation Method

**Figure 4:**
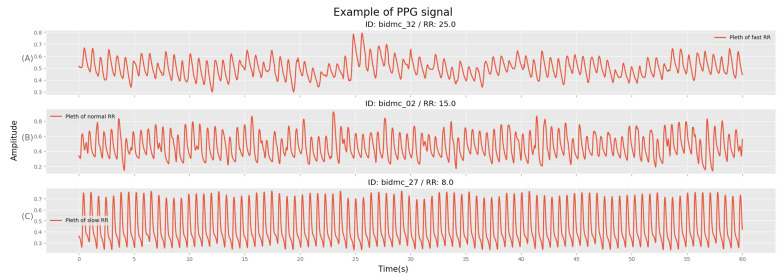
Examples of BIDMC patients’ 1 min PPG signals according to the breathing group. (**A**) Rapid breathing (>20 rpm), (**B**) normal breathing (12 to 20 rpm), (**C**) slow breathing (<12 rpm). Each example’s baseline wandering, amplitude modulation, and frequency modulation are related to their RRs. Thus, while subject (**A**) shows a dynamic signal movement, (**B**,**C**) show relatively constant movement.

To evaluate the models for each experiment, the test datasets were organized into four different groups: slow breathing group (<12 rpm), normal breathing group (12 to 20 rpm), rapid breathing group (>20 rpm), and all. The reason for this classification is that the RR is a significant vital sign that is associated with various diseases, and the PPG signal can vary accordingly (Figure 4), affecting the performance of models that estimate *RR* [42]. The performance of the model was evaluated using the mean absolute error (MAE), which calculates the error between the true and estimated values, for training, validation, and testing across all experiments conducted for each dataset.

(1) $$MAE=\frac{1}{N}\overset{N}{\underset{i=1}{\sum }}\left|RR_{true}^{i}-RR_{est}^{i}\right|$$

In the above formula, $RR_{true}^{i}$ and $RR_{est}^{i}$ represent the *i*-th actual *RR* and its corresponding estimated *RR*. *n* represents the size of the dataset. To evaluate and compare each result, we applied Min–Max normalization to all data, which replaces all values with values between 0 and 1.

### 2.6. Statistical Analysis

All statistical analyses were performed using the Numpy and Scipy libraries supported by Python and visualized using Matplotlib [43,44,45]. Before proceeding with preprocessing and model training using in-hospital data, we statistically tested whether the collected PPG data had any differences according to gender and existence of the disease, and finally set the data that were suitable for model training. To test whether the collected signal data grouped by gender were equal to its variances and averages, the Bartlett test and independent two-sample *t*-test were performed. After the models were trained, each of them derived estimated RRs corresponding to the actual RRs using a PPG from the validation dataset. Using a difference between these two values, our study evaluated its error and standard deviation, and then expressed it as MAE ± SD (brpm) to compare their performances. A box plot was used to evaluate each model’s estimation tendency, estimated RR distribution, and the number of outliers. Furthermore, linear regression analysis was applied to assess each model’s correlation between the actual and estimated values, and to be more specific, Pearson’s correlation coefficient (R) was also utilized to indicate the quantitative correlation between both observations. In addition, a Bland–Altman plot [46] analysis was exploited to confirm the agreement between them. Our study considered a level of significance for comparative analysis and testing as *p* < 0.05.

## 3. Results

From June 2022 to July 2022, a total of 100 patients admitted to the SICU of our institution were subjected to PPG and RR data collection and outcome analysis, including 56 males and 44 females. The mean age was 67.5 years, and the mean SOFA score on the day of admission to the SICU was 2.5 (range 0–6). A respiratory history of COPD or asthma was observed in seven patients (7%), and the most common diagnosis at admission was malignancy (54 patients; 54%), followed by non-cancerous lesions including ulcer perforation, pan-peritonitis, and bowel obstruction (28 patients; 28%). There were no differences in the baseline characteristics and disease profiles between the genders. (Table 1) As a result of the comparative analysis of the PPG data by gender, it was determined that the signal data of the two groups showed equal variance and consistency in the model training as the data did not show significant differences.

The data used in the study were first split into training data and testing data. The testing data were extracted in proportion to the three breathing groups, which were slow breathing (five subjects), normal breathing (five subjects), and rapid breathing (five subjects) for our own dataset, and one subject, three subjects, and three subjects for BIDMC, respectively. The reason why only one slow breathing subject was selected as the testing data in BIDMC was that there were only two slow breathing subjects in the entire BIDMC data, so we wanted to use the data of at least one subject for training. The training data were then split into a training dataset and validation dataset according to the 5-fold cross-validation method. Table 3 summarizes the number of samples for each dataset.

**Figure 5:**
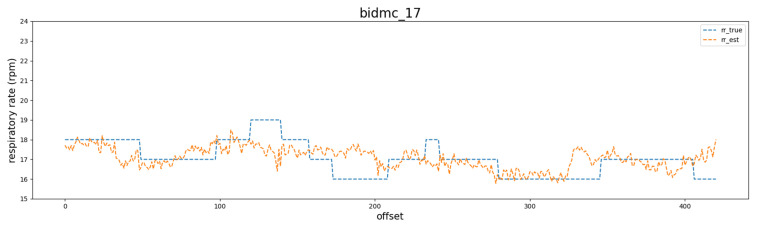
Example of sample RR estimation. The blue dotted line is the actual RR and the orange dotted line is the estimated RR.

Based on the above data, we ran the experiments described in the Experiments subsection and obtained the following results. Figure 5 shows the RR of the BIDMC validation dataset subject (bidmc_17), which was estimated using the Dilated ResNet model.

### 3.1. Training Model Using Self-Collected Dataset

All seven models introduced above were trained on our dataset, and the performances of the trained models were evaluated using the validation dataset. Table 4 summarizes these results. Next, an unseen dataset of each respiratory group was input to each model, and the results are shown in Table 5.

The results of evaluating the performance of the model trained with the self-collected dataset and the validation dataset are shown in Table 4. In Table 5, which shows the evaluation of RespNet’s unseen data, we can see that RespNet has the best performance on the same dataset as the trained data (in bold). We can also see that overall, most of the models have the best estimation performance for the normal breathing group within the dataset (in bold). The difference in the MAE between our dataset and CapnoBase within the same model is a maximum of 10.5005 and a minimum of 2.5712.

### 3.2. Training Model Using BIDMC Dataset

The performances of the seven models trained on BIDMC were evaluated on the validation dataset and are shown in Table 4. The models were also trained on the unseen dataset of each breathing group, and the results are shown in Table 6.

The performance evaluation on the BIDMC validation dataset in Table 4 shows that Bian’s ResNet, Ravichandran’s RespNet, and our proposed Dilated ResNet model perform well compared to the LSTM-based models. The MAE of these three models is around 1.27, while the MAE of the LSTM-based models is around 1.68. This behavior is also evident in Table 6, which shows the respiration rate estimation results of the models using the unseen dataset from various datasets. In this table, we again see that the three models perform best in alternating groups of datasets (in bold). We also see that when we drill down into each dataset by group, the best performing group is generally the normal breathing group. This is true across all seven models.

**Figure 6:**
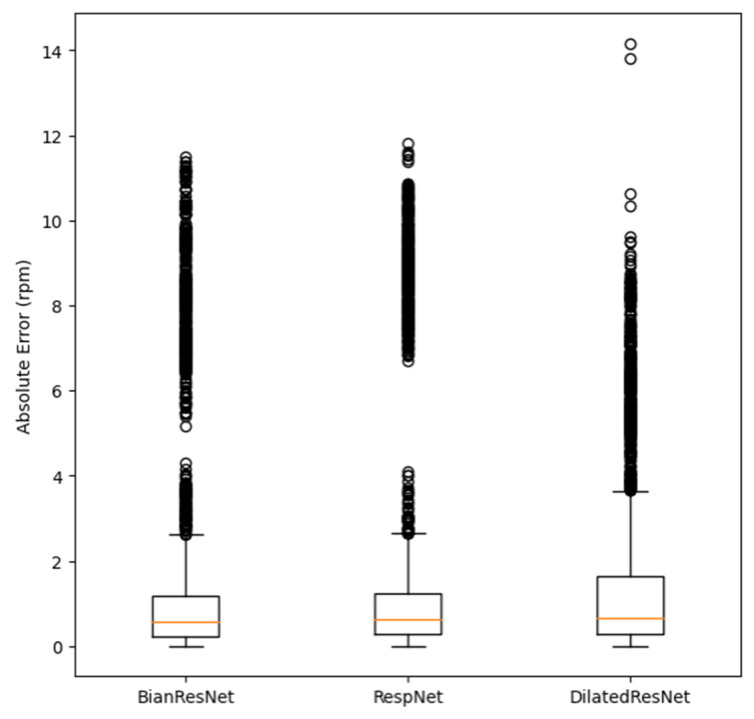
Comparison of the absolute error between two models. The box plot shows five values: minimum, lower quartile (25%), median (50%), upper quartile (75%), and maximum. Bian ResNet model (left) approximately shows 0.0004, 0.2362, 0.5843, 1.1879, and 2.6125 for each. RespNet (center) approximately shows 0, 0.2770, 0.865, 0.6393, 1.2240, and 2.6418 for each. Dilated ResNet (right) approximately shows 0.0009, 0.2823, 0.6503, 1.6312, and 3.6290 for each.

We wanted to check the results of the three most prominent models using the BIDMC validation dataset and the boxplot in Figure 6. The y-axis of the figure shows the absolute error (rpm), and the lowest error among the three models is obtained by RespNet, which is close to zero. The model with the lowest median is BianResNet, with a value of 0.5843. On the other hand, the model with the highest median is Dilated ResNet, with a value of 0.6503. Among the three models, Dilated ResNet has the fewest outliers for respiration rate estimation, with 454 outliers (13.48%) out of 3368 total data samples. Conversely, the model with the most outliers is BianResNet, with 531 outliers (15.77%). Furthermore, this paper calculated the Pearson correlation coefficient (PCC) between the estimated RR and the actual RR of each of the three models using the BIDMC validation dataset. As a result, BianResNet does not show a correlation between the estimated RR and actual RR at −0.0519 (*p* < 0.01). For RespNet, there is a weak negative correlation between the estimated and actual values at −0.3211 (*p* < 0.01). On the other hand, for Dilated ResNet, there is a weak positive correlation between the estimated and actual values at 0.5069 (*p* < 0.01).

### 3.3. Testing Model’s Robustness in Different SNRs

To test the robustness of the three models in the above experiments, we input the signals of different SNRs to compare and evaluate the results (Table 7).

According to the result, when the SNR is 10 db, BianResNet has the lowest error among the three models with an MAE of 2.2513, while our proposed Dilated ResNet model has the highest error with an MAE of 4.1142. However, in all other cases, we can see that our proposed model performs the best as it obtained values of 0.6303, 0.6417, and 0.8123 (in bold). However, the overall RR estimation error consistently increased as the SNR decreased in our experiment.

## 4. Discussion

In our study, we can see that RespNet and CNNLSTM perform better than the other models in Table 5, and BianResNet, RespNet, and Dilated ResNet perform better in Table 6. We believe this is because these models extract the spatial–spectral feature of the signal, which is often used in various PPG analysis studies [19,47]. Models that focus on temporal feature extraction, such as BiLSTM, also occasionally perform well using the temporal features of the signal, but more consistently perform well when using convolution and taking the shapes of previous signals and adding them together, such as ResNet.

In Table 5 and Table 6, we can confirm that the normal breathing group has the best results for all models in most cases. This is because of the group’s imbalance in the dataset that we used to train the model. This also can be seen in Table 3, which shows that both datasets have most of the data in the normal breathing group and have the least data in the slow breathing group. In the case of BIDMC, about 85% of the dataset consisted of data from the normal breathing group, which is a larger number than the other groups. The slow breathing group data, on the other hand, is only 3% of the dataset. Understanding this explains why the normal breathing group performs well in most cases. The reason for the better performance of the model trained on the BIDMC dataset compared to our dataset in Table 4 can be understood by expanding on the following: there are much more normal breathing data to train on, and the validation data are dominated by data from the same breathing class.

Using the BIDMC validation dataset, we compared each model’s estimates of RRs to the actual RRs to see if they were correlated. If the models overestimated the normal RR, i.e., if they had less error in estimating the actual RR, their estimates were positively correlated with the actual RR. However, BianResNet and RespNet produced either a negative correlation or no correlation of −0.0519 (*p* < 0.01) and −0.3211 (*p* < 0.01), respectively. This is likely due to the imbalance in the respiratory data, as discussed above. A negative correlation means that the straight-line output based on the actual and estimated values descends downward, and since BIDMC is almost dominated by normal breathing data, a negative slope may occur when the model estimates data belonging to the slow breathing group with higher estimated values than the actual values. On the other hand, in the case of Dilated ResNet, we confirmed a weak positive correlation of 0.5069 (*p* < 0.01). This means that the RR estimates, using the current signal data, are not highly correlated with the actual values, which points to the limitations of the current preprocessing.

To further consider that motion artifacts from patients in clinical practice can affect PPG-based RR estimation, we also confirmed the robustness of the three best-performing models to input signals of different SNRs, as shown in Table 7. The results show that Dilated ResNet has the highest estimation error at 10 db and the lowest errors are seen in the rest of the models. However, while the other models illustrate a gradual increase in error as the SNR decreases (i.e., as the shape of the input signal becomes more distorted), our model shows a sharp increase in error. This suggests that our model is more sensitive to noise that affects morphological features, as mentioned above. To improve this in future work, we would like to introduce regularization techniques to prevent our model from overfitting with the existing features. We would also like to use filters that are flexible to changes in the signal, such as adaptive filters [48], in preprocessing to increase the robustness of the overall model to various noises.

To estimate the RR, we filtered out only the 0.1–0.4 frequency band signals from the PPG that are likely to be associated with it. Also, it was processed to obtain a low-frequency signal from the PPG that contains information about movement due to respiration. At the same time, it was an attempt to exclude information such as the heartbeat. Many studies suggested various frequency bands [16,20,22,23]. In addition, in this paper, we resampled the input signal to reduce the computation of the model, in the same way as Bian [25]. Although this preprocessing allowed us to better focus on the data that we wanted to study, it caused a loss of information and the deformation of a signal in the original data. Such limitation has the potential of degrading the model’s performance and weakening correlations. In our future research, we should be aware of these points to improve the accuracy of RR estimation and more thoroughly investigate the appropriate frequency bands to remove unnecessary noise and capture more information for the estimation. Alternatively, it would be interesting to see and discuss the results of inputting such data without any information-losing preprocessing, taking advantage of the DL model’s ability to analyze the signal. This may be one way to improve the accuracy of RR estimation by applying preprocessing layers of noise filtering and signal detrending to PPG to overcome the existing manual de-modulation method, as an advantage of deep learning models is that they can convolve various filters and signals into PPG, which is difficult for humans to process. However, improving the reliability of these results remains a challenge. Although there are various attempts at Explainable AI [49,50], this is also an area for future research to improve the reliability of respiration rate estimation using PPG. It is possible to provide some evidence for the reliability of the result if the convolution filters, frequency filters, or detrending functions initialized in the DL process are adjusted to be more specific to RR estimation and placed in layers. The variety of factors that are present in a patient is another limitation that weakens the model’s performance. In our study, such components—diseases (e.g., hypertension, atrial fibrillation, etc.), interventions (e.g., vasopressor, ventilation, etc.), and other external influences—that modulate the PPG signal were not considered. Thus, in future work, we would like to study these characteristics, categorize patients, and confirm the model’s RR estimation performance for each factor.

Checking the box plot in Figure 6, we confirmed that Dilated ResNet has the highest standard deviation and median in errors among the three models. This suggests that our proposed model may be affected by various hyperparameters, as shown in Table 2, by applying a convolutional layer. Thus, future research will not only reduce the error of the proposed model but also clarify and study the causes of such deviations. To improve our model, it is necessary to generalize the model by applying regularization techniques such as L2 regularization, Dropout [51], replacing the RespBlock inner layer of the Dilated ResNet model to reduce RR estimation bias, and collecting additional data to provide the model with numerous signal patterns. A signal quality index (SQI) algorithm should also be added to reduce RR estimation error. The SQI algorithm is a technique that is intended to assess the signal and exclude signals with noise, which affect the training of the DL model in the preprocessing stage. Various algorithms, such as the skewness-based method, F1-score-based method, entropy-based method, machine learning-based method, etc., have been proposed to assess the quality of the PPG [16,52,53,54,55]. If we improve the quality of the PPG signal using an appropriate SQI algorithm that fits the PPG-derived DL model that we implemented for RR estimation in the follow-up study, we expect to estimate a more precise RR.

In Table 5 and Table 6, the difference in RR estimation resulting from the breathing rate group in the testing dataset is an obvious limitation that needs to be improved. To overcome this, in future research, we will fully utilize public datasets, but at the same time, try to configure our dataset to evenly test patients with different breathing rates and develop it into a public dataset. To carry this out, we are planning to carefully organize the database schema and data collection environment subject to a large data collection group and to collect the data over a long period, including the subject’s demographic information (e.g., age, gender, weight, etc.), medical record (e.g., whether they underwent surgery, infusion drug time, diagnosis, etc.), and various physiological signals (e.g., ECG, etCO_2_, etc.). Furthermore, by improving the methodology of this study, which collected data using only fingertips, we will collect PPG data from various body sites such as the earlobe or foot of a patient, analyze the measurements to compare the accuracy of the RR estimation between sites, and analyze the validity and association of PPG-derived RR estimation AI models according to the patient’s underlying disease and functional level. We expect to propose detailed guidance that is capable of sophisticated application according to the patient’s clinical status and measurement environment for the PPG-derived RR estimation AI model.

## 5. Conclusions

In conclusion, RR estimation using PPG-derived DL models is still challenging and has many limitations. Larger datasets, a model structure design, and preprocessing specialized in spatial–temporal feature extraction for the estimation are required. However, as the validation results in Table 4 show, if there are equal amounts of data from various breathing groups to train, we expect that the DL models, including our Dilated ResNet model, can achieve better results than the current ones.

## Figures and Tables

**Table 1:** The demographic characteristics and disease profiles of enrolled participants.

Variables	Total (*n* = 100)	Male (*n* = 56)	Female (*n* = 44)	*p*-Value
Age, years, mean ± SD * (range)	67.5 ± 14.2 (23–89)	66.7 ± 12.3 (23–89)	69.8 ± 16.9 (25–85)	0.680
SOFA score, mean ± SD (range)	2.5 ± 1.4 (0–6)	2.4 ± 1.4 (0–5)	2.6 ± 1.5 (0–6)	0.610
Underlying disease, *n* (%)				
Hypertension	27 (27)	14 (25)	13 (29.5)	0.828
Diabetes mellitus	25 (25)	14 (25)	11 (25)	1.000
COPD/asthma	7 (7)	4 (7.1)	3 (6.8)	1.000
Diagnosis, *n* (%)				
Malignancy	54 (54)	30 (53.6)	24 (54.5)	1.000
Non-cancerous lesion	28 (28)	16 (28.6)	12 (27.3)	1.000
Trauma	10 (10)	5 (8.9)	5 (11.4)	0.798
Miscellaneous	8 (8)	5 (8.9)	3 (6.8)	0.891

* SD = Standard Deviation.

**Table 2:** Hyperparameters for Bayesian optimization.

Hyperparameters	Range of Values	Selected Value
Number of RespBlocks ($N_{blk}$)	1~5	4
Kernel size of conv layer inside the RespBlock ($kernel_{blk}$)	2~5	2
Dilation rate of conv layer inside the RespBlock ($D_{blk}$)	1~5	3
Kernel size of conv layer for downsample ($kernel_{dwn}$)	2~4	3
Number of filters multiplied by power of two ($filters_{c}$)	4~10	8
Stride size of average pooling ($S_{c}$)	2~4	2
Units of first dense layer ($n_{den}$)	20~100	86

**Table 3:** Number of samples (subjects) for each dataset.

Datasets	Training and Validation				Test			
	Slow	Normal	Rapid	Total	Slow	Normal	Rapid	Total
Our Dataset	695	3314	1603	5612 (85)	190	466	240	896 (15)
BIDMC	421	15,575	2107	18,103 (43)	238	2201	508	2947 (7)
CapnoBase	–	–	–	–	3226	1978	269	5473 (13)

**Table 4:** Validation results of models trained using our dataset.

Models	MAE * ± SD ** (Our Dataset)	MAE * ± SD ** (BIDMC)
ResNet [25]	3.5316 ± 0.9043	1.2708 ± 0.3157
RespNet [26]	**3.1268 ± 0.6363**	1.2712 ± 0.3160
LSTM [27]	4.2924 ± 0.3952	1.6817 ± 0.2884
CNNLSTM [27]	4.2341 ± 0.4326	1.6908 ± 0.2837
BiLSTM [27]	4.2652 ± 0.4251	1.6882 ± 0.2933
Attention-based BiLSTM [27]	3.3355 ± 0.6971	1.6786 ± 0.2959
Dilated ResNet (Proposed)	3.3462 ± 0.6406	**1.2628 ± 0.2697**

* MAE = Mean Absolute Error; ** SD = Standard Deviation.

**Table 5:** Test results of models trained using our dataset.

Models	Our Dataset				BIDMC				CapnoBase			
	Slow	Normal	Rapid	Total	Slow	Normal	Rapid	Total	Slow	Normal	Rapid	Total
ResNet [25]	9.9752 ± 1.5697	2.6454 ± 2.0624	4.9341 ± 3.2360	**4.7612 ± 3.5064**	8.8698 ± 1.0238	2.7396 ± 1.5286	**1.8436 ± 0.8517**	2.7878 ± 1.8114	13.5344 ± 1.5362	6.9389 ± 2.1891	5.0811 ± 2.0314	9.8733 ± 3.0953
RespNet [26]	**3.5736 ± 2.0598**	**1.0024 ± 0.8990**	**4.5054 ± 2.9862**	4.9710 ± 3.2987	**6.6429 ± 3.1177**	9.1253 ± 3.9763	7.6840 ± 3.4102	8.9610 ± 4.1444	10.3029 ± 2.8448	9.0569 ± 1.4897	4.0897 ± 2.7380	11.5029 ± 3.8307
LSTM [27]	8.4881 ± 1.7116	3.0996 ± 1.6859	7.6986 ± 2.6673	5.4811 ± 3.1962	7.5067 ± 0.8415	**1.4029 ± 1.0809**	4.5474 ± 1.3353	**1.9855 ± 1.8139**	8.4744 ± 1.3914	3.1285 ± 1.9427	9.4211 ± 2.2129	6.5935 ± 3.0963
CNNLSTM [27]	8.0270 ± 1.7053	2.9366 ± 1.6073	8.0196 ± 2.6785	5.4094 ± 3.2448	7.1984 ± 0.8437	1.4271 ± 0.9911	4.9163 ± 1.3366	2.0367 ± 1.7949	**8.2459 ± 1.3921**	**3.0204 ± 1.8485**	9.6652 ± 2.2235	**6.4369 ± 3.0591**
BiLSTM [27]	8.4308 ± 1.7452	3.0698 ± 1.6718	7.7657 ± 2.6872	5.4867 ± 3.1987	7.4542 ± 0.8477	1.4032 ± 1.0747	4.6366 ± 1.3353	1.9872 ± 1.8121	8.4888 ± 1.3929	3.1096 ± 1.9285	10.0251 ± 2.2301	6.5795 ± 3.0941
Attention-based BiLSTM [27]	10.2409 ± 3.8196	4.4942 ± 3.0273	5.0869 ± 3.6495	7.1307 ± 3.9726	9.7364 ± 1.8992	4.4007 ± 1.7957	2.0115 ± 1.4983	4.2134 ± 2.4242	11.6212 ± 1.5833	6.4968 ± 2.2511	9.9008 ± 2.3863	9.7832 ± 3.3481
Dilated ResNet (Proposed)	8.1238 ± 2.1597	2.6191 ± 1.7300	5.1061 ± 2.5550	9.7765 ± 5.2752	8.4836 ± 1.6618	13.1615 ± 4.9493	2.1325 ± 1.3682	13.1983 ± 5.2574	14.9673 ± 2.5736	11.0462 ± 2.5060	3.3288 ± 1.7036	14.0638 ± 4.4238

**Table 6:** Test results of models trained using BIDMC dataset.

Models	Our Dataset				BIDMC				CapnoBase			
	Slow	Normal	Rapid	Total	Slow	Normal	Rapid	Total	Slow	Normal	Rapid	Total
ResNet [25]	**6.7551 ± 1.4659**	2.3388 ± 1.5861	10.2008 ± 3.7638	5.2620 ± 4.3497	**5.1753 ± 0.9849**	**1.9299 ± 1.7501**	8.1591 ± 1.3136	**2.3000 ± 1.4898**	**3.9111 ± 1.4059**	2.8848 ± 2.1277	9.2696 ± 2.0520	3.3907 ± 3.2020
RespNet [26]	7.3596 ± 1.5644	**1.9156 ± 1.4486**	8.7616 ± 3.6743	4.4259 ± 3.7224	7.1553 ± 1.2752	2.1594 ± 1.1806	6.3923 ± 0.8528	3.0716 ± 1.8902	6.4438 ± 1.4510	**2.1066 ± 1.2562**	9.6627 ± 1.9590	5.2284 ± 2.9006
LSTM [27]	8.3830 ± 1.4564	2.5204 ± 1.7610	6.7114 ± 3.2051	4.5045 ± 3.2727	7.6302 ± 0.7488	2.4339 ± 1.6054	3.4213 ± 0.6928	3.0237 ± 2.0134	8.6937 ± 1.3914	3.2512 ± 2.0568	9.1971 ± 2.2106	6.7515 ± 3.1394
CNNLSTM [27]	8.3352 ± 1.4555	2.5152 ± 1.7407	6.8162 ± 3.2058	4.5053 ± 3.2677	7.5736 ± 0.7489	2.4258 ± 1.5640	3.5066 ± 0.6838	3.0262 ± 1.9795	8.6084 ± 1.3961	3.2184 ± 2.0228	9.2106 ± 2.1942	6.7124 ± 3.1280
BiLSTM [27]	8.3728 ± 1.4567	2.5197 ± 1.7594	6.7144 ± 3.2055	4.5045 ± 3.2725	7.6295 ± 0.7460	2.4334 ± 1.6034	3.4170 ± 0.6934	3.0237 ± 2.0119	8.6904 ± 1.3916	3.2534 ± 2.0568	9.2453 ± 2.2108	6.7539 ± 3.1393
Attention-based BiLSTM [27]	8.5053 ± 1.4586	2.5759 ± 1.8162	6.5746 ± 3.2057	4.5154 ± 3.2677	7.7224 ± 0.7387	2.4917 ± 1.6578	3.2839 ± 0.7083	3.0555 ± 2.0589	8.8453 ± 1.3939	3.4019 ± 2.0630	9.1195 ± 2.2234	6.8935 ± 3.1338
Dilated ResNet (Proposed)	7.5543 ± 1.9206	1.9894 ± 1.2824	**5.3631 ± 3.2921**	**4.3177 ± 3.3040**	5.8661 ± 0.8277	2.3702 ± 2.1687	**2.5696 ± 1.3980**	2.6526 ± 2.2229	9.7006 ± 1.4092	4.1819 ± 2.1613	**8.3740 ± 2.4213**	7.6322 ± 3.1464

**Table 7:** Test result of models in different SNRs.

Models	Original	20 db	15 db	10 db
ResNet [25]	0.7203 ± 0.2655	0.7304 ± 0.2729	1.4932 ± 0.4728	**2.2513 ± 0.4843**
RespNet [26]	0.7122 ± 0.3290	0.7265 ± 0.3328	2.0491 ± 0.4456	2.5544 ± 0.3368
Dilated ResNet (Proposed)	**0.6303 ± 0.3197**	**0.6417 ± 0.3258**	**0.8123 ± 0.3744**	4.1142 ± 0.4804

## Data Availability

The datasets used and analyzed during the current study are available from the corresponding authors upon reasonable request.

## Abbreviations

The following abbreviations are used in this manuscript.

AI	Artificial Intelligence
APACHE	Acute Physiology And Chronical Health Evaluation
DL	Deep Learning
ECG	Electrocardiogram
ICU	Intensive Care Unit
MAE	Mean Absolute Error
MEWS	Modified Early Warning Score
PPG	Photoplethysmogram
RR	Respiratory Rate
SD	Standard Deviation
SICU	Surgical Intensive Care Unit
SOFA	Sequential Organ Failure Assessment
SQI	Signal Quality Index
